# Genomic analysis of acid tolerance genes and deciphering the function of *ydaG* gene in mitigating acid tolerance in *Priestia megaterium*

**DOI:** 10.3389/fmicb.2024.1414777

**Published:** 2024-06-20

**Authors:** Darshana Sharma, Purna Bahadur Chetri, Vipin Ranga, Subhajit Sen, Bidyut Kumar Sarmah, Madhumita Barooah

**Affiliations:** ^1^DBT—North East Centre for Agricultural Biotechnology, Assam Agricultural University, Jorhat, Assam, India; ^2^Department of Agricultural Biotechnology, Assam Agricultural University, Jorhat, Assam, India

**Keywords:** *Priestia megaterium*, general stress protein, genome analysis, growth curve, acid tolerance

## Abstract

Adverse environmental conditions, such as acid stress, induce bacteria to employ several strategies to overcome these stressors. These strategies include forming biofilms and activating specific molecular pathways, such as the general stress response (GSR). The genome of *Priestia megaterium* strain G18 was sequenced using the Illumina NextSeq 500 system, resulting in a *de novo* assembly of 80 scaffolds. The scaffolded genome comprises 5,367,956 bp with a GC content of 37.89%, and was compared to related strains using the MiGA web server, revealing high similarity to *P. megaterium* NBRC 15308 and *P. aryabhattai* B8W22 with ANI scores of 95.4%. Phylogenetic and ribosomal multilocus sequence typing (rMLST) analyses, based on the 16S rRNA and ribosomal protein-encoding alleles, confirmed close relationships within the *P. megaterium* species. Functional annotation identified 5,484 protein-coding genes, with 72.31% classified into 22 COG categories, highlighting roles in amino acid transport, transcription, carbohydrate metabolism, and ribosomal structure. An in-depth genome analysis of *P. megaterium* G18 revealed several key genes associated with acid tolerance. Targeted inactivation of the *ydaG* gene from SigB regulon, a general stress response gene, significantly reduced growth under acidic conditions compared to the wild type. qRT-PCR analysis showed increased *ydaG* expression in acidic conditions, further supporting its role in acid stress response. Microscopic analysis revealed no morphological differences between wild-type and mutant cells, suggesting that *ydaG* is not involved in maintaining cellular morphology but in facilitating acid tolerance through stress protein production. This research contributes to understanding the molecular mechanisms underlying acid tolerance in soil bacteria, *P. megaterium*, shedding light on potential applications in agriculture and industry.

## 1 Introduction

It is evident that a significant portion of soil bacteria exists in a non-growing or starvation state for a substantial duration, primarily due to their exposure to various adverse environmental conditions in their natural habitat (Roszak and Colwell, 1987; Morita, 1988; Gray et al., 2019; Metze et al., 2023). To endure environmental stress, bacteria have developed unique strategies of fundamental importance to maintain cell viability and regrowth in the environment, which is crucial for their survival where growth conditions are restricted due to natural factors. The evolution of intricate adaptational networks enables bacterial survival and growth under stressful environments (Volker et al., 1994; Yang et al., 2016; Tan et al., 2022). Numerous neutrophilic bacteria, including *Escherichia coli* and *Salmonella* spp. employ various mechanisms to maintain pH homeostasis, including growth restriction strategies to survive extreme pH conditions (Audia and Foster, 2003; Foster, 2004). Growth and survival of the bacteria under stress involve alterations in metabolism, transportation pathways, and cellular structure. The cell membrane plays a significant role in acid tolerance in response to pH drop, as evidenced by changes in the membrane’s fatty acid profile (Quivey et al., 2001; Guan and Liu, 2020; Xu et al., 2020).

Bacteria exposed to various environmental stress conditions have evolved multiple intricate molecular pathways that are interconnected to ensure their survival under stressful environments. One of the best examples is the formation of biofilm (Pedrido et al., 2013; Vlamakis et al., 2013). Several studies have demonstrated that *Bacillus subtilis* displays an immediate and effective cellular reaction known as a general stress response (GSR) when exposed to diverse environmental stress (Rodriguez Ayala et al., 2020). This response involves the immediate activation and transient expression of about 150 general stress proteins (GSP) regulated by the transcription factor called SigB (Binnie et al., 1986; Hecker et al., 2007; Losick and Pero, 2018). The whole genome sequencing of *B. subtilis* strain 168 (Kunst et al., 1997) revealed the involvement of almost 200 genes under SigB regulon using the advancement of omics and post-genome strategies (Price, 2000a,b; Hecker et al., 2007; Nicolas et al., 2012). Comparative genomic and proteomic analysis of wild-type and mutant SigB strain of *B. subtilis* under various stressed and unstressed conditions facilitate the identification of the significant number of GSP assigned to SigB regulon (Hoper et al., 2005). A substantial portion of these proteins have proven to have biochemical functions. The expression of SigB regulon induced under certain stress conditions inhibits the growth rate of bacteria; this might be to protect the cells from various stresses that would otherwise compromise the survival of the cell survival in the future (Price, 2000a; Hecker et al., 2007; Nicolas et al., 2012).

*Priestia megaterium* is classified as an aerobic, spore-forming Gram-positive, neutrophilic bacterium. It is widespread across diverse environments but most commonly associated with soil. Its capability to grow at broad temperatures, ranging from 3°C to 45°C, and its capacity to metabolize diverse carbon sources makes it a prime candidate for industrial applications (Vary et al., 2007). Besides industrial purposes, *P. megaterium* has other applications in promoting plant growth activity, including protection against plant pathogens (Kildea et al., 2008; Nguyen et al., 2011). Recently *Bacillus megaterium* has recently been moved to the new genus *Pristia* and now known as *Pristia megaterium* (Gupta et al., 2020). Our earlier studies have reported that the growth of *P. megaterium* in an acidic medium (pH 4.5) induces the expression of several acid-tolerance genes (Goswami et al., 2018). Among these genes, *ydaG* expression increases 10-fold in an acidic medium (Goswami et al., 2018). Several studies have suggested that activation of SigB regulon induces the expression of GSP that controls enzymes involved in DNA protection, protein synthesis under stress, antibiotic resistance, heat resistance, cell wall protection, cold resistance, etc. (Price, 2000a,b; Hecker et al., 2007; Nicolas et al., 2012). The role of GSP in acid tolerance in *P. megaterium* has not been explored yet. We have previously reported that a network of mechanisms confers acid tolerance in *P. megaterium* (Nicolas et al., 2012; Goswami et al., 2018).

Here, we reported the whole genome sequence analysis of *P. megaterium* to identify the genes involved in acid tolerance and further explored the role of the general stress response gene *ydaG* from SigB regulon in conferring acid tolerance in *P. megaterium*.

## 2 Materials and methods

### 2.1 Bacterial strains, plasmid vectors, culture media, and primers

*Priestia megaterium* G18 (Goswami et al., 2017) and TOP10 *E. coli* bacterial strain were used in this study. The TOP10 *E. coli* strain was used as the host for plasmid transformation and propagation and was routinely cultured in a complex medium. To facilitate the targeted gene inactivation in *P. megaterium* G18 we utilized pMUTIN4 (BGSC, OSU, United States) as an integration vector. This vector contains *ermAM* that encodes for rRNA adenine N-6-methyltransferase for the selection of Gram-positive bacteria, that provides resistance to erythromycin (0.3 μg/mL). It also carries *bla* gene (β-lactamase) for selection in ampicillin medium (100 μg/mL), which is necessary for plasmid maintenance in *E. coli*. *Priestia megaterium* G18 was cultivated either in minimal media (MM) or Nutrient Broth (NB, Himedia, India), with necessary antibiotics as needed. The *E. coli* was cultured in Luria Broth (LB, Himedia, India) in the presence or absence of required antibiotics. The pH of the media was adjusted using Hydrochloric acid (HCl) if needed. Agar powder (Difco, United States) at 15 g/L was used to solidify the media when required. Ampicillin at 100 μg/mL concentration was used to select the TOP10 *E. coli* strain. *Priestia megaterium* G18 was selected in media supplemented with erythromycin and trimethoprim. The Bacterial growth was measured at OD_600nm_ using a Spectroquant Pharo 300 spectrophotometer (Merk, Germany). Plasmids and the bacterial strains utilized in this investigation are mentioned in previous studies (Goswami et al., 2022). Oligonucleotide primers and the sequence utilized in our study are shown in Table 1. All the oligonucleotide primers used in this study were ordered from Integrated DNA Technology (IDT, www.idtdna.com).

**Table 1:** Oligonucleotides primers and their sequence used in the study.

Sl. No	Primer name	Sequence (5′-3′)	Description
1	ermAM-F	GAA CAA AAA TAT AAA ATA TTC TCG	ermAM gene present in the pMUTIN4 vector, used to detect the insertion of pMUTINydaG into the genome of transformed cells.
2	ermAM-R	TCC TCC CGT TAA ATA ATA GAT AAC T	
3	ydaG-F	CGCAAGCTTCCCACTCTCGCTATATGACATTC	Used to amplify internal fragment of ydaG gene and its cloning into pMUTIN4 vector for inactivation of ydaG gene.
4	ydaG-R	CGCGGATCCATTTGGACCGTCAAACCATTC	

### 2.2 DNA sequence library preparation

For whole-genome sequencing, genomic DNA was isolated from *P. megaterium* G18 using standard CTAB (cetrimonium bromide) and phenol: chloroform extraction method followed by RNase A treatment and purification (Wilson, 2001). Isolated DNA was quantified using NanoDrop (SPECTROstar Nano BMG Labtech). After quantification, a pair-end sequencing library was prepared from the QC passed genomic DNA sample using Illumina TrueSeq Nano DNA Library Prep Kit (Illumina, United States). Then, the QC passed paired-end library was sequenced on the NextSeq 500 system (Illumina, United States). After sequencing, the raw data was generated for adapter trimming and genome assembly (Sen et al., 2020).

### 2.3 Genome assembly

FastQC v0.11.9 tool available at https://www.bioinformatics.babraham.ac.uk/projects/fastqc/ was used to check the quality of the raw reads. Adapters and low-quality reads were trimmed using the TrimGalore v0.6.7 software available at https://github.com/FelixKrueger/TrimGalore. The high-quality reads were assembled into contigs using the ABySS tool (Simpson et al., 2009). ABySS is a *de novo*, parallel, and short paired-end reads assembler that builds de Bruijn graphs across various k-mer values. Among these, a k-mer value of 127 was the optimal choice based on the estimated assembly size, number of contigs, and N50 value. The generated genome assembly was refined using four rounds of polishing with bwa v0.7.17 (Li and Durbin, 2009), samtools v1.13 (Li et al., 2009), and Pilon v1.22 (Walker et al., 2014). The ribosomal multilocus sequence typing (rMLST) analysis was conducted on the assembled genome for species identification based on the count of ribosomal MLST alleles (Jolley et al., 2012). To identify the most closely related bacterial species based on Average Nucleotide Identity (ANI) and genome coverage, the polished assembly was subjected to the TypeMat tool available at the MiGA web server (Rodriguez et al., 2018). The complete genome of *P. megaterium* strain NBRC 15308 (NCBI reference ID: NZ_CP035094; genome size: 5,343,009 bp) was used as a reference to scaffold the contigs of *P. megaterium* G18. The RagTag tool was employed for scaffolding and improving genome assembly. Quality assessment of the assembly was performed using QUAST (Gurevich et al., 2013), and the genome coverage was calculated using bwa v0.7.17 (Li and Durbin, 2009) and samtools (Li et al., 2009). CheckM v1.2.2 was utilized to estimate genome completeness and potential contamination of the assembled genome (Parks et al., 2015).

### 2.4 Annotation and comparative genomic analysis

Genes on the scaffolded sequences were annotated using Prokka v1.14.6 (Seemann, 2014) and the COGclassifier v1.0.5 (Shimoyama, 2022) tools. A local sequence blast of protein-coding sequences with unknown functions was performed for functional annotation analysis. An annotated GenBank (GBK) file obtained from Prokka was uploaded on the Proksee web server to visualize features associated with the circular genome of *P. megaterium* G18 (Grant et al., 2023). Acid tolerance genes of microbes identified by literature mining at PubMed were mapped on the circular genome in Proksee. The assembled genome of *P. megaterium* G18 was compared with genomes of closely related species *Priestia megaterium* strain NBRC 15308 and *Priestia aryabhattai* strain B8W22 using Proksee.

### 2.5 Phylogenetic analysis and taxonomy classification

The scaffolded DNA sequences were screened for 16S rRNA fragments using the ContEST16 web server (Lee et al., 2017). Subsequently, the identified sequence was searched against the nr database using the blastn program at NCBI. The 16S rRNA sequences of closely related bacterial species were retrieved from the NCBI database. Phylogenetic relationships between the 16S rRNA sequences were studied using the neighbor-joining and maximum likelihood methods. The neighbor-joining tree was generated in the MEGA v11.0.13 tool with 1,000 bootstrap replicates (Kumar et al., 2018). The maximum composite likelihood statistical model, transition and transversion substitution, and complete deletion of gaps were also chosen as important parameters for constructing the phylogenetic tree. The maximum likelihood tree was generated using the Hasegawa-Kishino-Yano model with the following parameters: gamma distributed with invariant sites (G + I) rates, complete deletion of gaps, and 1,000 bootstrap replicates (Kumar et al., 2018). A large-scale pan-genome analysis was performed on the eight selected genomes from different species of the genus *Priestia* using the Roary v3.13.0 package (Page et al., 2015). For this, first, GFF (General Feature Format) files were produced using the Prokka v1.14.6 tool. A multiple sequence alignment of the nucleotide sequences of the core genes was generated using the MAFFT tool in Roary. Next, Roary’s FastTree v2.1.11 tool with the GTR (Generalized Time-Reversible) model was used to construct a phylogenetic tree from the large aligned sequences (Price et al., 2009).

### 2.6 Molecular modeling of ydaG protein

The model of ydaG was built using SWISS-MODEL web server (Waterhouse et al., 2018). Three-dimensional (3D) coordinates from the “General Stress Protein 26 (GSP26)” protein (PDB ID: 3EC6, resolution 1.6 Å) were utilized to prepare the structural model of ydaG. The best 3D model was selected based on parameters such as QMEAN score, percent identity, percent coverage, and clash score. Next, the quality of the ydaG model was assessed using the Ramachandran plot in the SWISS-MODEL web server. The 3D model was visually inspected and compared with the template GSP26 protein in the Schrödinger PyMOL v3.0.0. tool.^1^ A superimposed figure of ydaG with GSP26 was prepared using PyMOL.

### 2.7 Targeted inactivation of *ydaG* gene

The targeted inactivation of the *ydaG* gene was carried out using a pMUTIN4 (Vagner et al., 1998) integration vector purchased from BSGC, United States. This vector has ori (origin of replication) functioning in *E. coli* and is nonfunctional in Gram-positive bacteria. It was created for the targeted inactivation of genes in *Bacillus* sp. The recombinant plasmid was incorporated into the host chromosome by cloning the internal sequence of the target ORF into the vector’s cloning site and transforming it into the suitable host cell. This leads to a disruption of the *ydaG* gene, and the resulting mutant (s) can be selected on nutrient agar plates enriched with 0.1 μg/mL of erythromycin. The NucleoSpin Microbial DNA kit (Macherey–Nagel, Germany) was used to isolate the genomic DNA from *P. megaterium* G18 in accordance with the manufacturer’s instructions. For amplification of the internal region of the *ydaG* gene, the sequence of the *P. megaterium* ATCC14581 *ydaG* gene was obtained from the gene database,^2^ and the oligonucleotide primers were designed from the sequence using Primer Quest tool^3^ containing HindIII and BamHI restriction site in the forward and reverse primer, respectively. Gene amplification was carried out according to the GoTaq® DNA polymerase protocol (Promega, United States) in a final reaction volume of 50 μL containing 20 pmol of each primer, 2 U Taq DNA polymerase, and 50 ng of genomic DNA of *P. megaterium* G18. The reaction mixture was placed into a thermal cycler (Applied Biosystems, United States) with the following program: 30s of initial denaturation at 94°C, 30s of annealing at 60°C, 30s of extension at 72°C, and 7 min of final extension at 72°C. HindIII and BamHI restriction enzymes were used to digest the amplified *ydaG* fragment and the pMUTIN4 vector. Then, using a NucleoSpin® Gel and PCR Clean-up kit (Macherey–Nagel, Germany), the digested products were purified. Using T4 DNA ligase (Takara, Japan), the purified ydaG fragment was ligated to the digested pMUTIN4 to produce the recombinant pMUTydaG plasmid. The ligated product was chemically transformed into the competent *E. coli* TOP10 cells (Invitrogen™ United States), and the recombinant plasmid pMUT*ydaG* was isolated using Wizard® Plus SV Minipreps DNA Purification System kit (Promega, United States). As reported earlier, the purified recombinant plasmid was used to transform *P. megaterium* G18 protoplast (Chang and Cohen, 1979).

### 2.8 Phenotypic analysis of *ydaG* mutant

The bacterial colonies observed in NA plates were further subcultured for three more generations. Those bacterial colonies displaying antibiotic resistance on the plate are considered mutant and characterized for their growth pattern and acid susceptibility. The wild type and the mutant were freshly cultured in NB media until the OD_600_ reached 1.0. The cultures containing around 6 log CFU/mL were selected for inoculation into NB and minimal media of varying pH (4.5 and 7.0) and kept at 37°C with continuous rotation at 150 rpm for 12 h. To monitor the growth, cells at different pH were serially diluted, and cultures were plated on the NA media. The colonies that emerged on the plates were counted and reported as log CFU/mL.

### 2.9 Mutants verification by *ermAM* gene amplification using PCR

To verify the integration of pMUTydaG into the chromosome of acid-susceptible isolates, PCR amplification of the *ermAM* gene was conducted, utilizing genomic DNA from the mutants as a template. Genomic DNA from the mutant strains was extracted using a NucleoSpin Microbial DNA isolation kit (Macherey–Nagel, Germany). The primer pair (ermAM-F & ermAM-R) was custom-designed with Primer Quest software based on the nucleotide sequence of the pMUTIN4 vector (GeneBank accession number: AF072806.1) to target the ermAM gene. Amplification of the ermAM gene was carried out using the designated forward and reverse primers in a thermal cycler (2,720 Thermal cycler, Applied Biosystem®, United States) following the GoTaq® DNA polymerase protocol (Promega, United States). The PCR reaction mixture, totaling 50 μL, comprised 20 pmol of each primer, 2 U of Taq DNA polymerase, and 50 ng of genomic DNA. The PCR protocol included initial denaturation at 94°C for 3 min, followed by 35 cycles of denaturation at 94°C for 30 s, annealing at 60°C for 1 min, and extension at 72°C for 45 s, with a final extension step at 72°C for 7 min. The amplified products were then analyzed using 1% agarose gel electrophoresis and visualized under UV light. Positive and negative controls were included using the pMUTIN4 plasmid and genomic DNA of *P. megaterium* G18, respectively. Acid-susceptible isolates that tested positive for the *ermAM* gene were chosen for further investigation.

### 2.10 Validation of *ydaG* gene expression in wild-type and mutant *Priestia megaterium* G18 cells through qRT-PCR

The mutation and *ydaG* gene expression levels were confirmed through qRT-PCR. The cells were grown freshly in NB media till the OD_600_ reached 1.0 to perform RT-PCR analysis of the wild type and the mutant. Subsequently, these cultures were used to inoculate the minimal media with varying pH (4.5 and 7.0) and incubated at 37°C with continuous shaking at 150 rpm for 8 h. The cells were pelleted by centrifugation, and the total RNA was extracted using a Nucleospin RNA Mini kit following the manufacturer’s protocol (Macherey–Nagel, Germany). Next, a Goscript cDNA synthesis kit (Promega, Madison, United States) was used to synthesize cDNA from the total RNA. The single-stranded cDNA was used to perform Real-time PCR using GoTaq qPCR Master Mix (Promega, Madison, United States) containing 50 ng cDNA template 10 pmol of each primer in a total volume of 20 μL according to the manufacturer’s protocol. The real-time PCR was performed in three biological replicates in Applied Biosystems, United States, and the 16S rRNA gene was used as a reference gene.

### 2.11 Growth curve analysis of wild-type and mutant (Δ*ydaG*) *Priestia megaterium* G18

The growth curve characteristics of both wild-type and Δ*ydaG* mutant *P. megaterium* G18 cells were monitored in the minimal medium at varying pH (pH 4.5,7.0, and 8.5). Initially, both the mutant and wild-type cells were cultured in MM media at pH 7.0 until the OD_600_ reached 1.0. Subsequently, 1,000 μL of both mutant and wild-type cultures were transferred to the MM media with a pH of 4.5. The growth curve was constructed by measuring the OD_600_ value at specific time intervals and comparing them with the wild-type and mutant *P. megaterium* G18 cells grown at pH 7.0, 8.5, and 4.5.

### 2.12 Microscopic analysis

A field emission scanning electron microscope (FESEM, Carl ZEISS, SIGMA, Germany) was used to study any morphological alteration under acidic stress. Both Δ*ydaG* mutant cells and wild-type *P. megaterium* G18 cells were cultured in minimal media with a pH of 7.0 until an OD_600_ of 1.0 was reached. In separate conical flasks, 1 mL of each wild type and mutant culture were transferred to new minimum media (pH 4.5) and allowed to grow for 1 h. Following Gram staining, the cells were removed and examined under a microscope, and the morphologies of WT and mutant cells cultured under various circumstances were analyzed.

## 3 Results

### 3.1 Whole genome sequence assembly and general features

**Figure 1:**
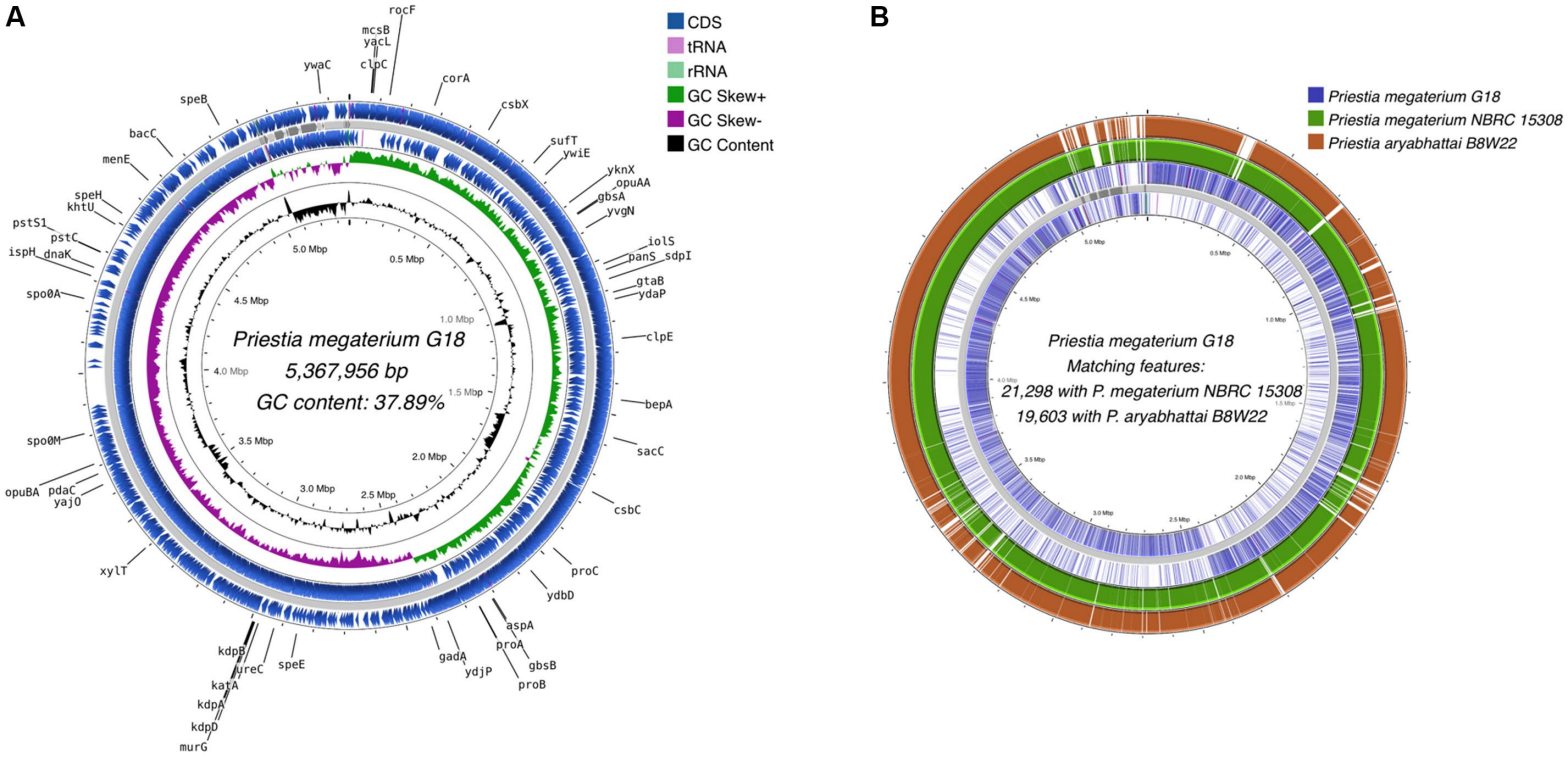
Whole-genome assembly map of *Priestia megaterium* G18. **(A)** The circular assembled genome shows the location and arrangement of the annotated protein-coding sequences (CDS). The names of CDS involved in stress responses and acid tolerance are shown in black. Starting from the outermost ring to the center: CDS (blue), tRNA (pink), and rRNA (light green) on the forward strand; assembled scaffolds (gray); CDS (blue), tRNA (pink) and rRNA (light green) on the reverse strand; a pattern of GC skewness (purple and green); and pattern of GC content (black). **(B)** Comparative genome analysis of *Priestia megaterium* G18 with the genomes of two closely related species. Starting from the outermost ring to the center: Genomic features of *P. aryabhattai* B8W22 (brown); genomic features of *P. megaterium* NBRC 15308 (green); genomic features on the forward strand of *P. megaterium* G18 (blue); assembled scaffolds (gray); and genomic features on the reverse strand of *P. megaterium* G18 (blue).

A total of 5,370,570 paired-end reads of length 150 bp were obtained when sequencing the genome of *P. megaterium* G18 using the Illumina NextSeq 500 sequencing platform. The raw reads were processed, and those with a Phred quality score of ≥25 and read length of ≥40 bp were selected for genome assembly. A total of high-quality 5,329,347 reads (99.23%) were *de novo* assembled using the ABySS tool, and a final assembly of 102 contigs was obtained. Closely related bacterial strains identified by the MiGA (Microbial Genomes Atlas) web server were *P. megaterium* NBRC 15308 and *P. aryabhattai* B8W22, both showed an Average Nucleotide Identity (ANI) score of 95.4%. A total of 80 scaffolds were obtained after chaining contigs together using information from the genome of the closely related *P. megaterium* NBRC 15308 (NCBI reference ID: NZ_CP035094). The general characteristics of the *P. megaterium* G18 genome at both contigs and scaffold levels are listed in Table 2. The scaffolded genome comprises 5,367,956 bp with an average genome-wide GC content of 37.89% (Figure 1A). Comparative analysis of genomic features between *P. megaterium* G18 and the two closely related species showed differences at several locations, suggesting the possible gene gain and/or loss of multiple genes, which may have evolutionary advantages for enhancing their competitiveness in an acidic environment (Figure 1B). The analysis of circular genomes further revealed a higher degree of homology between *P. megaterium* G18 and *P. megaterium* NBRC 15308 compared to *P. aryabhattai* B8W22 (Figure 1B). In Table 3, a comparison of the genomic features of *P. megaterium* G18 is presented. This includes genome size, GC content, number of contigs, number of scaffolds, N50, L50, number of CDS, genome coverage, and genome completeness for *P. megaterium* NBRC 15308 and *P. aryabhattai* B8W22, two closely related species based on the nucleotide-level genomic similarity. Since the genome of *P. aryabhattai* B8W22 is assembled at the contig level, information regarding scaffolds is unavailable.

**Table 2:** General features of the *Priestia megaterium* G18 genome at contigs and scaffolds levels.

Features	Contigs level	Scaffolds level
Genome size (bp)	5,366,228	5,367,956
GC percent (%)	37.88	37.89
Total number	102 contigs	80 scaffolds
Total number (with length ≥ 1,000 bp)	30 contigs	16 scaffolds
N50	1,145,649	5,045,504
L50	2	1
Number of CDS	5,485	5,484
Number of tRNAs	116	116
Number of rRNAs	24	24

**Table 3:** Comparison of genomic features of *Priestia megaterium* G18 with *P. megaterium* NBRC 15308 and *Priestia aryabhattai* B8W22.

Features	*P. megaterium* G18	*P. megaterium* NBRC 15308	*P. aryabhattai B8W22*
GenBank assembly ID	-	GCA_006094495.1	GCA_000956595.1
Genome size (bp)	5.36 Mb	5.7 Mb	5.1 Mb
GC percent (%)	37.89	38	38
Number of contigs/scaffolds	102/80	7/7	72/ NA
Contig N50/Scaffold N50	1.14 Mb/5.04 Mb	5.3 Mb/5.3 Mb	125.5 kb/ NA
Contig L50/Scaffold L50	2/1	1/1	14/ NA
Number of CDS	5,484	5,596	4,807
Genome coverage	298x	103x	17.8x
CheckM (completeness in %)	99.43	99.27	98.79
Contamination (%)	0.64	0.32	0.38

Data for *P. megaterium* NBRC 15308 and *P. aryabhattai* B8W22 were retrieved from the genome database at NCBI. NA, Not available.

### 3.2 Phylogenetic and ribosomal multilocus sequence typing analysis

**Figure 2:**
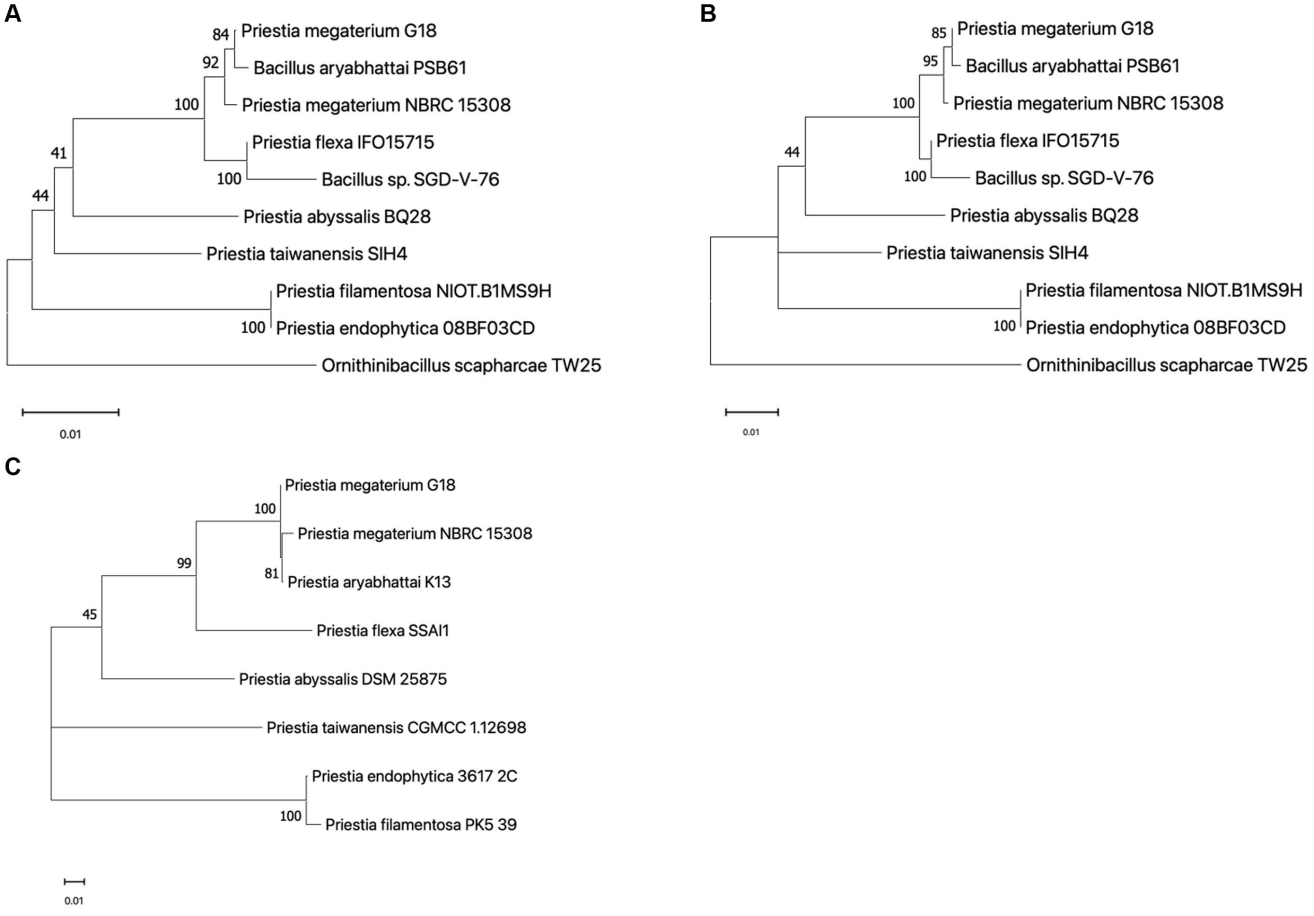
Phylogenetic analysis. **(A)** A neighbor-joining phylogenetic tree built with 16S rRNA sequences highlighting the position of *Priestia megaterium* G18 relative to other species within the genus. The following sequences were retrieved from the NCBI database (accession ID in brackets): *P. megaterium* G18 (predicted from the ContEST16 web server), *P. megaterium* NBRC 15308 (MH071135.1), *P. aryabhattai* PSB61 (HQ242774.1), *P. flexa* IFO15715 (NR_024691.1), *Bacillus* sp. SGD-V-76 (KF413434.2), *P. abyssalis* BQ28 (OM534577.1), *P. taiwanensis* SIH4 (OL377898.1), *P. filamentosa* NIOT.B1MS9H (OR623178.1), *P. endophytica* 088F03CD (KX146479.1), and *Ornithinibacillus scapharcae* TW25 (HQ171440.1). The tree was rooted with *O. scapharcae* TW25. Numbers at nodes are support values from 1,000 bootstrap replicates. **(B)** Maximum likelihood tree based on the Hasegawa-Kishino-Yano model with G + I parameters using 16S rRNA sequences from the abovementioned species. The numbers on each node correspond to the bootstrap support value. The tree was rooted with *O. scapharcae* TW25. **(C)** An unrooted maximum likelihood phylogenetic tree was generated using core-genome sequences from eight species of the *Priestia* genus. The following genomes were retrieved from the genome database at NCBI (GenBank ID in brackets): *P. megaterium* NBRC 15308 (GCA_006094495.1), *P. aryabhattai* K13 (GCA_002688605.1), *P. flexa* SSAI1 (GCA_022559225.1), *P. abyssalis* DSM 25875 (GCA_002019595.1), *P. taiwanensis* CGMCC 1.12698 (GCA_014638355.1), *P. endophytica* 3617_2C (GCA_003269955.1), and *P. filamentosa* PK5_39 (GCA_003600795.1). The numbers on nodes correspond to the bootstrap support value. Number 0.01 on the scale bar (bottom) represents one substitution in 100 bp.

The 1,552 bp long 16S rRNA sequence of *P. megaterium* G18 was computationally predicted using ContEST16 (Supplementary Table 1). It showed sequence identity ranging from 99.94 to 99.81% with the complete genomes of various strains of both *P. megaterium* (GenBank IDs: CP045272.1, CP049296.1, CP047699.1, CP069288.1 and CP058255.1) and *P. aryabhattai* (GenBank IDs: CP041519.1 and CP041516.1). Comparative 16S rRNA sequence analysis revealed that *P. megaterium* G18 was most closely related to *P. megaterium* NBRC 15308 (99.73%), *P. aryabhattai* PSB61 (99.55%), *P. flexa* IFO15715 (98.91%), and *Bacillus* sp. SGD-V-76 (98.11%). The *P. megaterium* G18 strain was found to form a cluster with *P. megaterium* NBRC 15308 and *P. aryabhattai* PSB61 in the neighbor-joining phylogenetic tree generated from the 16S rRNA dataset (Figure 2A). Analysis of the maximum-likelihood phylogenetic tree generated from the 16S rRNA sequences strongly confirmed the close relationship of *P. megaterium* G18 with *P. megaterium* NBRC 15308 and *P. aryabhattai* PSB61 (Figure 2B). In addition, a pan-genome-based maximum-likelihood phylogenetic tree of eight different species of the genus *Priestia* (*P. megaterium* G18, *P. megaterium* NBRC 15308, *P. aryabhattai* K13, *P. flexa* SSAI1, *P. abyssalis* DSM 25875, *P. taiwanensis* CGMCC 1.12698, *P. endophytica* 3617_2C, and *P. filamentosa* PK5_39) showed that *P. megaterium* G18 clusters with both *P. megaterium* NBRC 15308, *P. aryabhattai* K13 (Figure 2C). The pan-genome analysis revealed that out of all the identified genes in the eight species, 3,750 genes were common among *P. megaterium* G18, *P. megaterium* NBRC 15308, and *P. aryabhattai* K13. In contrast, only 177 genes were found to be common between *P. megaterium* G18 and its distantly related *P. flexa* SSAI1 species.

The ribosomal Multilocus Sequence Typing (rMLST) analysis identified 57 bacterial ribosomal protein-encoding alleles matching exactly with the corresponding *rps* genes in the assembled genome of *P. megaterium* G18 (Supplementary Table 2). Among these, 46 alleles encoding the ribosomal proteins supported the *Priestia* genus, while the remaining 11 alleles (*rpsA*, *rpsB*, *rpsD*, *rpsE*, *rpsT*, *rplB*, *rplD*, *rplI*, *rplO*, *rplT*, and *rplW*) were specifically predicted for the *P. megaterium* species. Thus, all the predicted alleles that matched with the *Priestia* genus and were specifically associated with *P. megaterium* provided strong support for the identification of a new strain of *P. megaterium*.

### 3.3 Functional annotation

**Figure 3:**
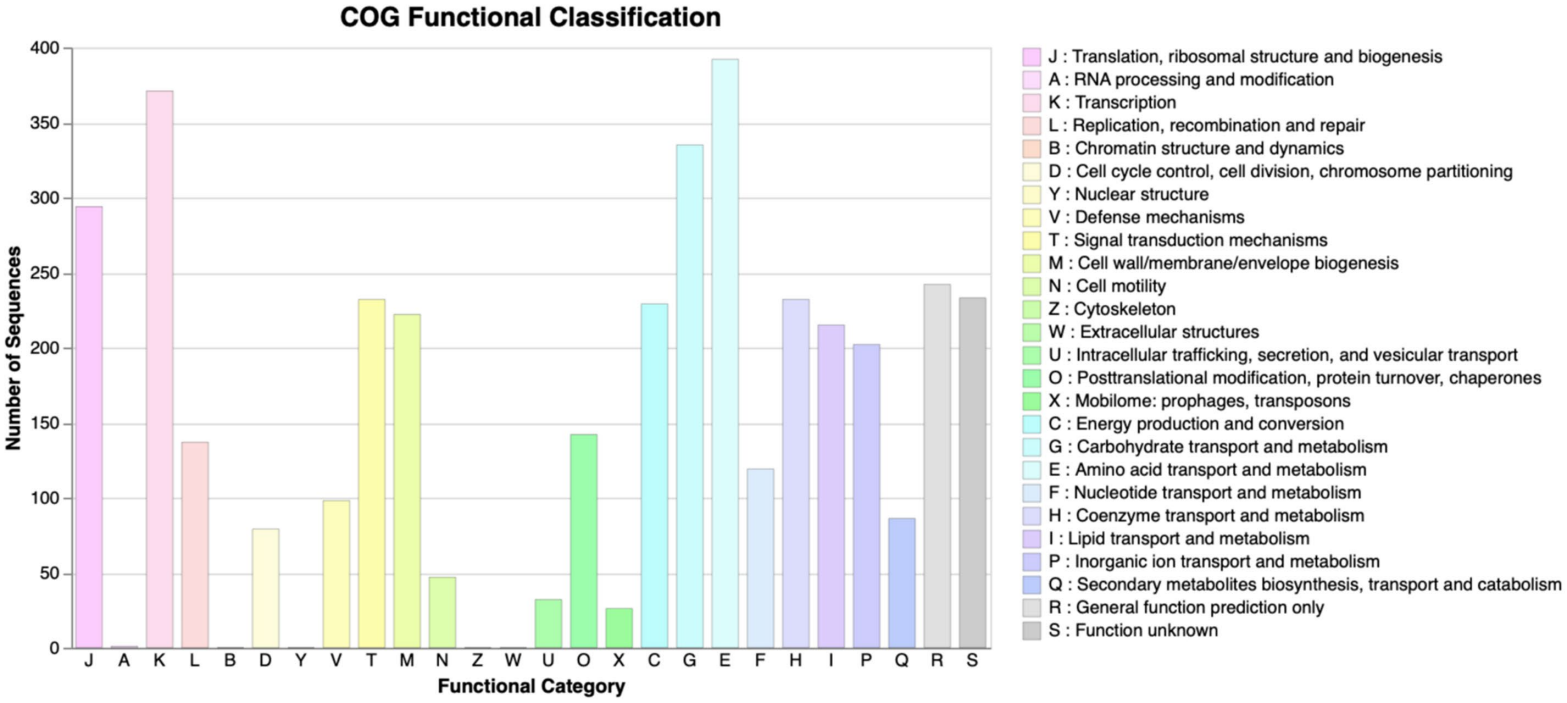
COG functional annotation. COG annotation of the proteins encoded by the predicted CDS in *Priestia megaterium* G18. All the CDSs were classified into 22 different functional categories. All 26 functional categories, each denoted by an alphabet, are indicated on the x-axis; the number of CDS in each category is mentioned on the y-axis.

**Figure 4:**
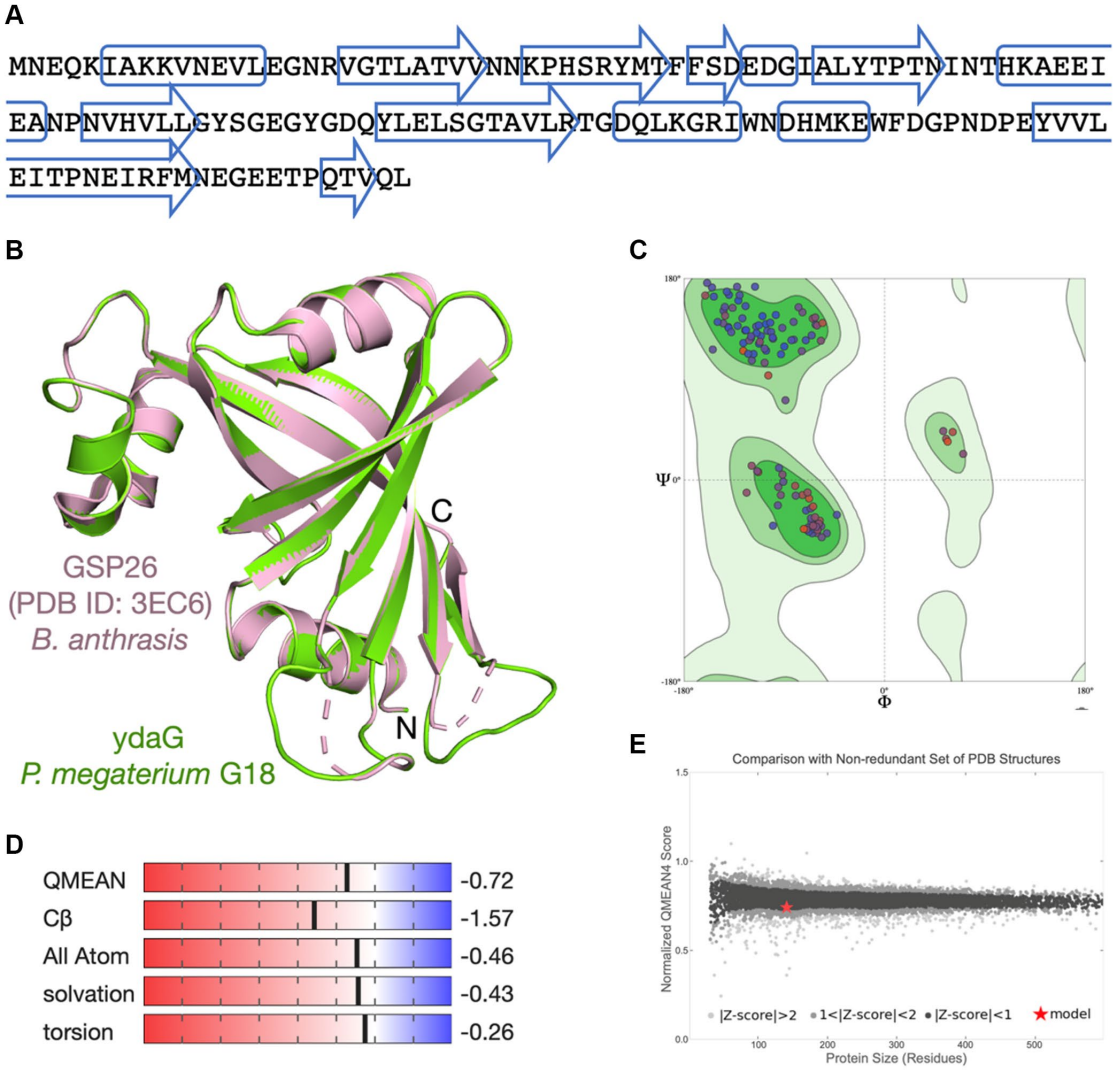
Molecular modeling of ydaG protein. **(A)** Amino acid sequence of the ydaG protein. The predicted secondary structure regions are shown in box (α-helix) and arrows (β-strand). **(B)** The ydaG model (in green) is superimposed on the GSP26 template structure (PDB ID: 3EC6) from *Bacillus anthrasis* (in pink). Three-dimensional coordinates of the GSP26 protein structure were used for molecular modeling of ydaG. **(C)** Ramachandran plot shows both the phi (ϕ) and psi (ψ) torsion angles adopted by the main chain of each amino acid in ydaG. **(D)** Scores associated with critical parameters used to assess the quality of the generated model are displayed. These parameters include QMEAN score, Cβ interactions, interactions between all atoms, solvation, and torsion angles. **(E)** The ydaG model is represented as a red star. The x-axis represents the number of amino acids in proteins, whereas the y-axis shows the “QMEAN” score. Each dot represents an experimentally determined structure. Dots with a QMEAN Z-score between 0 and 1 are in black color.

The scaffolded genome was predicted to contain 5,484 protein-coding DNA sequences (CDS), 116 tRNA sequences, and 24 rRNA operons. First, the Prokka tool was utilized to find functions of all the predicted CDS of *P. megaterium* G18. A total of 3,091 (56.36%) genes were annotated with a known biological function, while 2,393 (43.63%) were annotated as hypothetical proteins or proteins with uncharacterized function. Next, the COGclassifier tool was used to classify the predicted CDS into different functional categories, as defined by the Cluster of Orthologous Groups (COGs; Figure 3). Among the 5,484 CDS, a total of 3,966 (72.31%) CDS were assigned to 22 specific COG categories. The top four COG categories with the highest number of coding sequences were as follows: category E, amino acid transport and metabolism (9.88%); category K: transcription (9.35%); category G: carbohydrate transport and metabolism (8.45%); and category J: translational, ribosomal structure and biogenesis (7.41%). The COG “defense mechanisms” category contained 98 CDS (2.47%) of the predicted genes. A total of 233 (4.24%) CDS were categorized in the “function unknown” COG class. A blastx search of these CDS against the protein database of *P. megaterium* NBRC 15308 annotated function to 212 genes. All the 212 annotated CDS shared more than 80% identity with the sequences of *P. megaterium* NBRC 15308. The analysis results showed that two stress-related genes encoding “general stress protein 26 (GSP26),” initially predicted by the Prokka tool and assigned to the “function unknown” COG category, showed more than 98% sequence identity with proteins from the pyridoxamine 5′-phosphate oxidase (PNPO) family (NCBI accession IDs: WP_013055138.1 and WP_063671472.1). Blastp search of the 142 amino acids long GSP26 (see sequence in Figure 4A) against the non-redundant NCBI database showed 100% sequence identity with the *ydaG* protein of *P. megaterium* WSH-002 (GenBank ID: AEN89968.1). In addition, the annotated domain in the InterPro database indicated that the GSP26 protein belongs to the family of stress response and developmental protein (InterPro ID: IPR052917) and contains a ydaG FMN-binding split barrel domain according to the InterPro database (InterPro ID: IPR038725) and pyridoxamine 5′-phosphate oxidase-like domain according to the Pfam database (Pfam ID: PF16242). Both the FMN-binding protein and pyridoxine 5′-phosphate oxidase are known to contain the FMN-binding split barrel domain.^4^ Hence, based on these observations, we identify GSP26 as the ydaG protein. To understand the functional importance of individual amino acids, we performed a detailed structural analysis of the ydaG protein in *P. megaterium* G18.

### 3.4 Overall description of the three-dimensional structure of ydaG in *Priestia megaterium* G18

Since the function of a protein is determined by its three-dimensional (3D) structure, we aim to examine the arrangement of amino acids in the ydaG protein structure of *P. megaterium* G18. No experimental structure of the ydaG protein is available for species from the *Priestia* genus. We used a SWISS-MODEL-based molecular approach to study the structural details. A sequence search at the NCBI database suggested a total of 3 templates, showing that the percent identity fell in the range of 30 to 40%. The closest template to ydaG was identified as GSP26 in *Bacillus anthrasis* (PDB ID: 3EC6, resolution 1.6 Å), showed a sequence identity of 36.76%, 96% query coverage, 0.75 GMQE score, and a QMEAN score of 0.74 ± 0.05. The structural model of monomeric ydaG demonstrated a distinct PNPO-like fold (Figure 4B). A close inspection of the model revealed that amino acids important for protein folding were similar/identical to the template structure in most places. Quality assessment of the model revealed no steric clashes, and all the Cα-atoms were identified within the Ramachandran favored regions (Figure 4C). Other assessment parameters, such as QMEAN score, Cβ interactions, interactions between all atoms, solvation, and torsion angles, collectively indicated that the ydaG model exhibits high quality (Figure 4D). Comparison of the ydaG model with existing experimentally determined structures resulted in a plot between normalized QMEAN score and protein size (Figure 4E). In this plot, a value of |Z-score| < 1 was assigned to ydaG (indicated as a red star in Figure 4E), suggesting that the model contained a 3D fold very similar to the experimentally known structures and reflected a “native-like” structure. In terms of function, ydaG has been demonstrated as a crucial protein participating in the stress response mechanism in the Bacillus genus (Volker et al., 1994; Kunst et al., 1997). Structurally, ydaG is known for its ability to form a functional ABC transporter through dimerization with the ydbA protein (Lubelski et al., 2004).

### 3.5 Targeted inactivation of *ydaG* gene

PCR amplification of the internal fragment of the *ydaG* gene was performed with forward and reverse primers. The amplified product was cloned into the pMUTIN4 vector and transformed into the *E. coli* cells, and positive clones (pMUT*ydaG*) were selected (Supplementary Figure S1). The *P. megaterium* G18 was transformed with pMUT*ydaG* and was selected on NA media that contained erythromycin (0.4 μg/mL). The antibiotic-resistant cells appeared on the NA plate and were selected as pMUT*ydaG* transformants. The mutant cells were confirmed based on the culture traits and through PCR amplification of the inserted *ermAM* gene. The integration of the pMUTIN4 plasmid into the host chromosome occurs through a single recombination process between the *ydaG* gene locus and the homologous chromosomal locus. Since the pMUTIN4 vector contains *ermAM* gene, insertion of the vector into the host chromosomal DNA confers resistance to erythromycin in the mutant cells, resulting in selection into the growth medium containing erythromycin (Supplementary Figure S2). To further confirm the integration of pMUT*ydaG* into the host chromosome, a PCR reaction of the *ydaG* gene was performed with gene-specific primers. No amplification of the *ydaG* gene confirmed the successful integration of the pMUT*ydaG* vector into the gene locus as it disrupts the gene (Supplementary Figure S3). We also confirmed the mutation by PCR amplification of the *ydaG* gene using genomic DNA of wild-type and mutant cells grown at pH 7 and 4.5 (Supplementary Figure S4). Our observation showed that the *ydaG* gene amplified in the wild type at pH 7 and 4.5. However, in the case of mutant cells, the *ydaG* gene amplification did not occur due to the *ydaG* gene disruption by the pMUTIN4 vector.

### 3.6 Survivability of *Priestia megaterium* G18 mutant and wild-type cells at different pH (4.5, 7.0, and 8.5)

**Figure 5:**
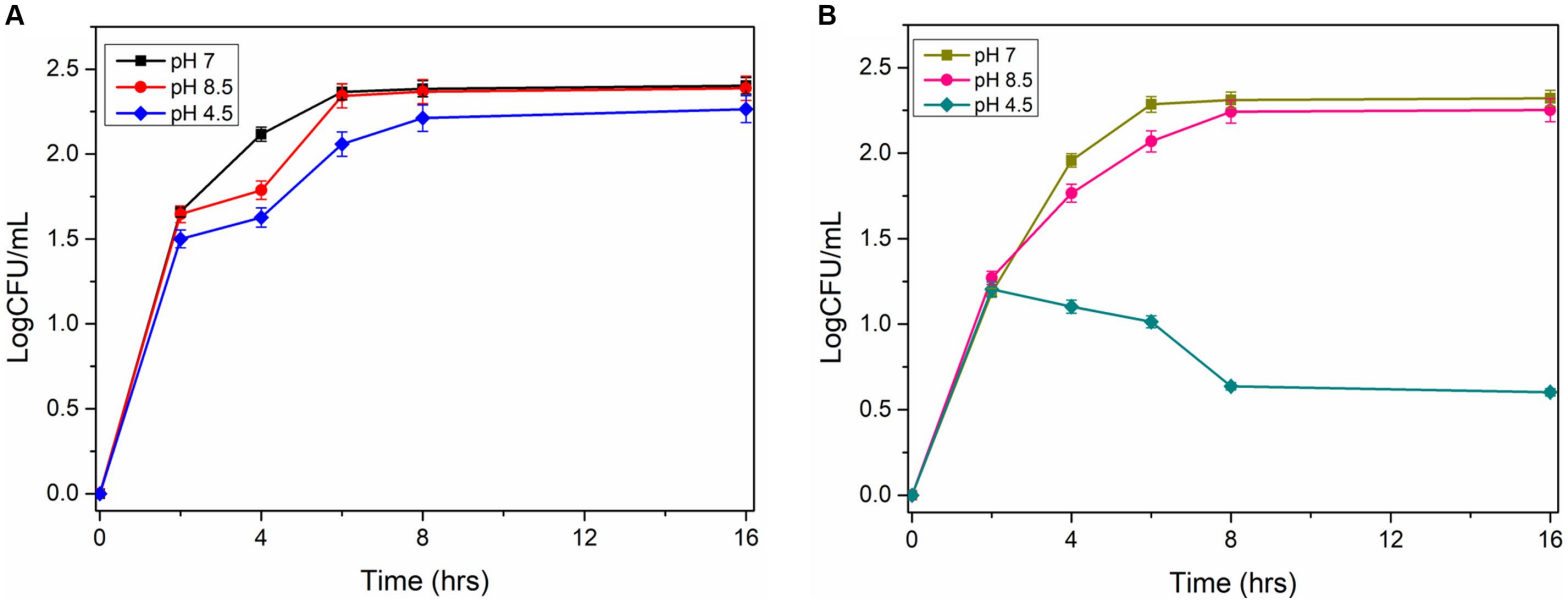
Survivability of *Priestia megaterium* G18 wild type and mutant at different pH (7.0, 4.5 and 8.5). **(A)** CFU Growth curve plot of *P. megaterium* G18 wild-type cells at pH 7 (black), pH 8.5 (red), and pH 4.5 (blue). **(B)** CFU Growth curve plot of *P. megaterium* G18 Δ*ydaG* mutant cells at pH 7 (Dark yellow), pH 8.5 (pink), and pH 4.5 (dark cyan).

The acid tolerance characteristics of the mutant and wild-type cells were analyzed in NB culture media at different pH (7, 4.5, and 8.5). The serially diluted bacterial culture was plated on the agar plate to check viable cell counts under normal and acidic pH conditions. The experiments revealed that wild-type cells grow normally (9.2 logCFU/ mL after 12 h) at pH 7 and pH 4.5. However, the mutant cells grow normally at pH 7, but the growth reduces significantly under acidic conditions (0.4–1 logCFU/mL after 12 h; Figures 5A,B). This observation demonstrated that the disruption of the *ydaG* gene reduces the colony-forming unit per mL under acidic conditions, reducing the acid tolerance capability of *P. megaterium* G18 cells.

### 3.7 Growth curve analysis of wild-type and mutant (Δ*ydaG*) *Priestia megaterium* G18

**Figure 6:**
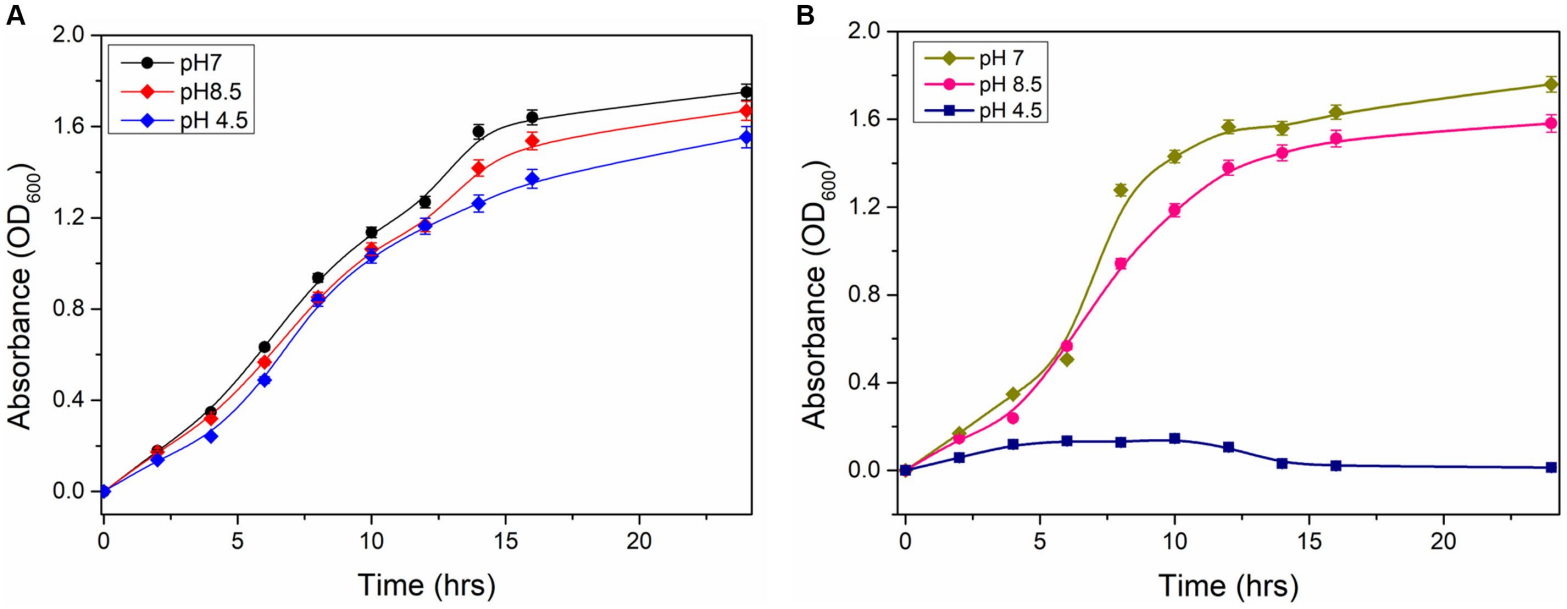
Growth properties of *Priestia megaterium* G18 wild type and mutant cells. **(A)** Growth characteristics of *P. megaterium* G18 wild-type cells at pH 7 (black), 8.5 (red), and 4.5 (blue), respectively. **(B)** Growth characteristics of *P. megaterium* G18 Δ*ydaG* mutant cells at pH 7 (dark yellow), 8.5 (pink), and 4.5 (navy), respectively.

The growth characteristics of wild type and Δ*ydaG* mutant of *P. megaterium* G18 under normal and acidic conditions were analyzed. Initially, cells were grown at pH 7.0 until the OD_600_ reached 1.0. The wild-type and mutant cells of different densities were transferred to pH 4.5 to analyze the acid tolerance capability. The wild-type cells were observed to grow normally at pH 7, and when pH was changed to 4.5, a lag phase of 2 h was observed, and after that, the exponential growth was achieved. In the case of Δ*ydaG* mutant cells, the growth was normal at pH 7. However, when the mutant cells were grown at pH 4.5, the growth of the cells was reduced significantly (Figures 6A,B). This confirms that the *ydaG* gene confers acid tolerance in *P. megaterium* G18, and the mutation of the *ydaG* gene increases the acid susceptibility of the bacteria.

### 3.8 qRT-PCR analysis of wild-type and mutant cells

**Figure 7:**
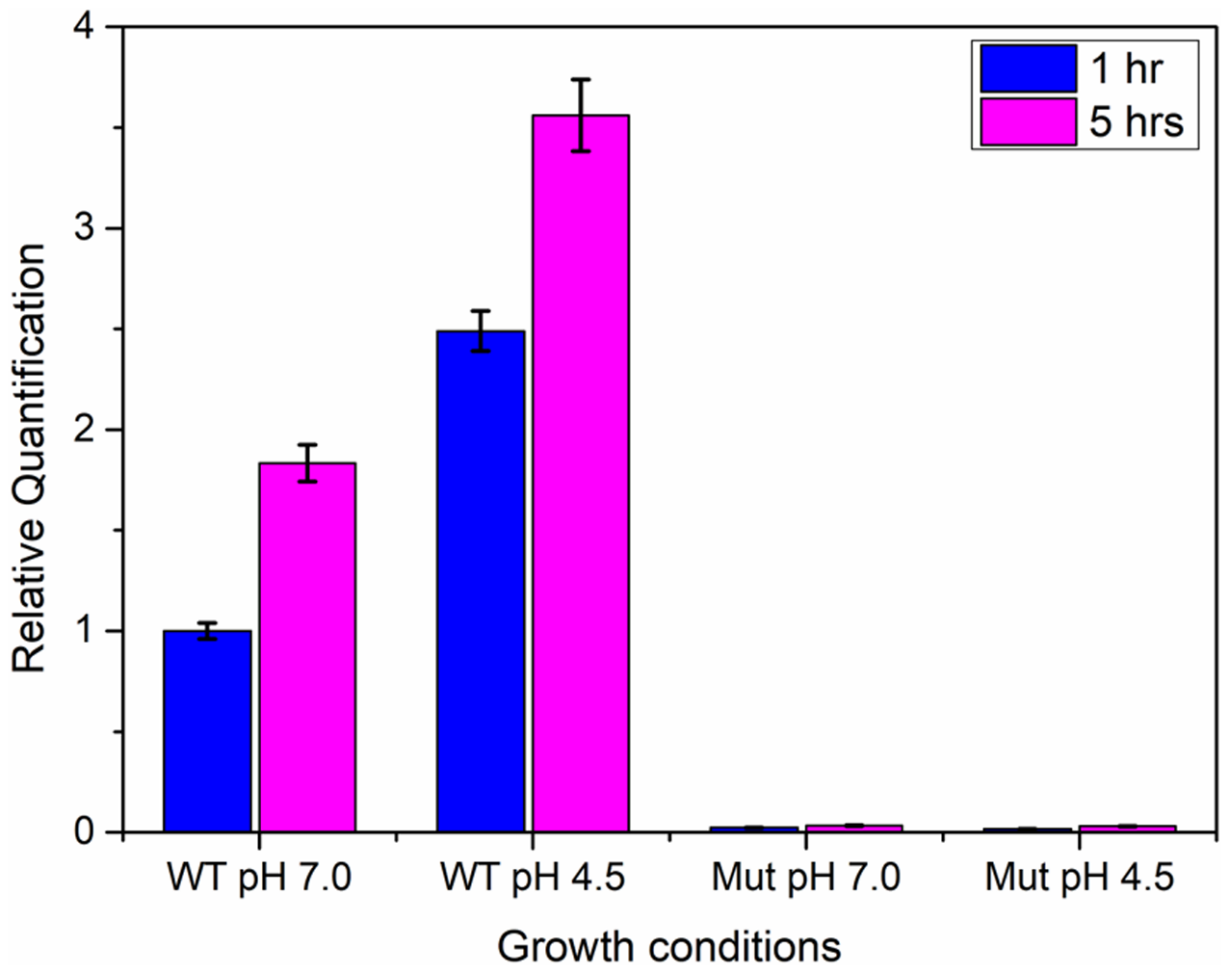
qRT-PCR analysis of *ydaG* gene expression in wild-type and mutant cells at pH 7 and 4.5 at two different time intervals (1 and 5 h).

The qPCR analysis revealed a two-fold increase in the expression of the *ydaG* gene in wild-type cells when grown at an acidic pH of 4.5 compared to neutral pH at both intervals (1 and 5 h). The mutant cells showed no expression at pH 4.5 and 7 due to disruption of the *ydaG* gene (Figure 7). The mutant cells could survive at pH 7 but could not survive at pH 4.5.

### 3.9 Microscopic analysis

**Figure 8:**
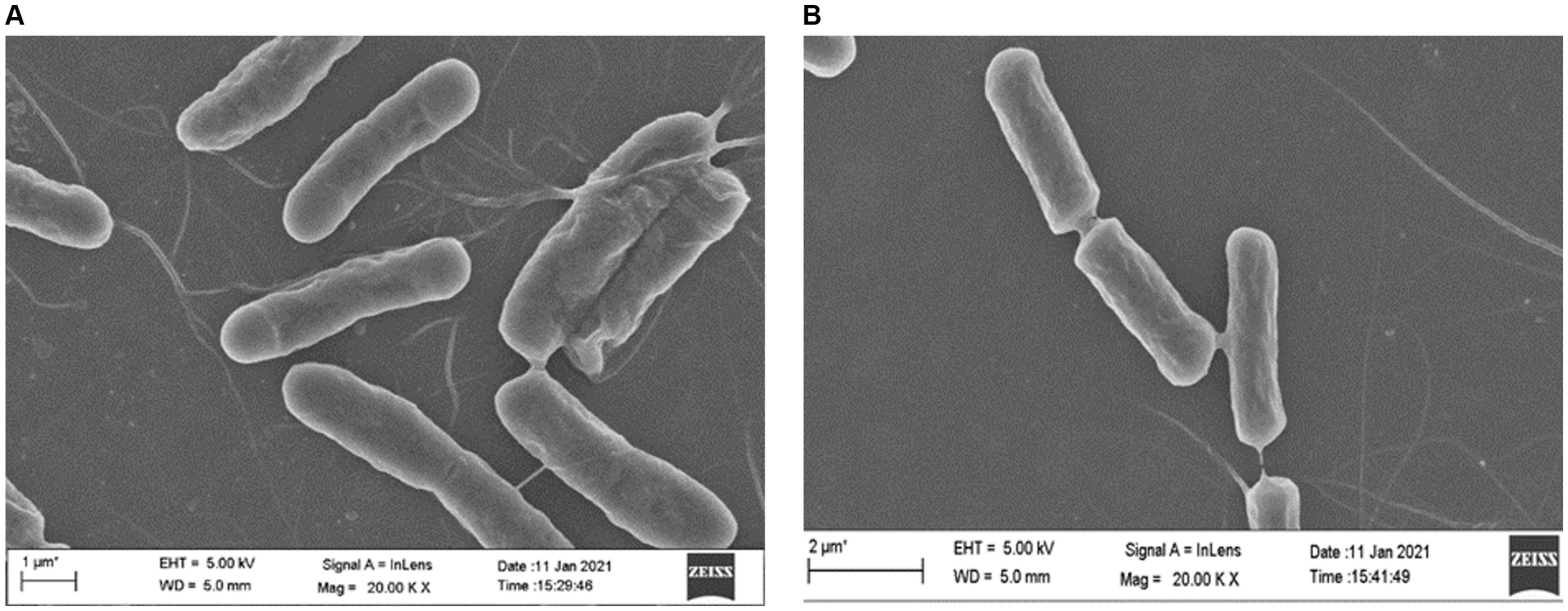
Microscopic analyses to examine the effect of acid (pH 4.5) on wild-type *Priestia megaterium* G18 and Δ*ydaG* mutant cell morphology. **(A)** Cell morphology of *P. megaterium* G18 wild-type cells grown at pH 4.5. **(B)** Cell morphology of Δ*ydaG* mutant cells grown at pH 4.5.

Field scanning electron microscopic examination of the wild type and the *ΔydaG* mutant in normal and acidic conditions revealed no morphological differences in wild type and mutant cells in acidic environments. This observation demonstrated that the *ydaG* gene does not have a role in maintaining cellular morphology (Figures 8A,B). It may have a different role in facilitating acid tolerance in bacteria by producing general stress protein, which helps bacteria overcome acidic stress.

## 4 Discussion

We explored the genomic makeup of acid-tolerant bacteria, particularly focusing on *P. megaterium* strain G18, isolated from acidic soils of Assam, India (Supplementary Figure S5). By employing high-throughput sequencing techniques, we deciphered the complete genomic information of *P. megaterium* G18 and compared it with closely related species. The Illumina high-throughput sequencing technique was employed to obtain the complete genomic information of *P. megaterium* strain G18. The assembled genome (5,367,956 bp) of *P. megaterium* G18 contained 80 scaffolds with an N50 of 5,045,504 bp. Compared to the two most homologous species, the genome of *P. megaterium* G18 was 5.34% larger than that of *P. aryabhattai* B8W22 (5,095,483 bp) and 6.58% smaller than that of *P. megaterium* NBRC 15308 (5,746,548 bp). The GenBank assembly IDs for *P. megaterium* NBRC 15308 and *P. aryabhattai* B8W22 are GCA_006094495.1 and GCA_000956595.1, respectively. Despite its close relation to *P. megaterium* NBRC 15308 and *P. aryabhattai* B8W22, significant differences were observed in genome size, number of protein-coding sequences, and ribosomal MLST analysis. The genome of *P. megaterium* G18 contained a total of 5,484 protein-coding sequences (CDS), which was 14.08% higher than the number of CDS found in *P. aryabhattai* B8W22 (4,807 CDS), but only 2% lower than that of *P. megaterium* NBRC 15308 (5,596 CDS). Comparing the 16S rRNA sequence of *P. megaterium* G18 with that of *P. megaterium* NBRC 15308 and *P. aryabhattai* B8W22 showed a 0.18% difference in sequence identity. While the differences were relatively small, observations on the genome size, number of CDS, and 16S rRNA sequence identity indicated that *P. megaterium* G18 is more closely related to *P. megaterium* NBRC 15308 than to *P. aryabhattai* B8W22. A conclusive bioinformatics analysis was needed to validate the closely related species to *P. megaterium* G18. Therefore, the ribosomal MLST analysis was adopted to search for the most highly conserved ribosomal alleles in the assembled genome of *P. megaterium* G18. In this analysis, *P. megaterium* NBRC 15308 showed full support for all the ribosomal alleles present in *P. megaterium* G18, while *P. aryabhattai* B8W22 showed support for 80.7%.

Functional annotation of different genes highlighted the prevalence of various stress response mechanisms, particularly those associated with amino acid transport and metabolism (9.88%), transcriptional regulation (9.35%), carbohydrate transport and metabolism (8.45%), and translational, ribosomal structure and biogenesis (7.41%). Amino acid transport and metabolism significantly minimize the acidic stress response in bacteria (Guan and Liu, 2020). According to Senouci-Rezkallah et al. (2011), amino acids raise pH levels during metabolism, making a variety of bacteria acid-tolerant. These systems are known as acid tolerance systems dependent on amino acids. It has been determined that the arginine deaminase (ADI) pathway plays a significant role in protecting certain bacteria from acid damage (Shabayek and Spellerberg, 2017). Different transcriptional regulators were also associated with acid tolerance in bacteria (Guan and Liu, 2020). Table 4 displays the stress response and acid tolerance genes of microbes identified through literature mining.

**Table 4:** List of stress response and acid tolerance genes identified through literature mining.

Sl. No.	Gene	Coding protein	Particular function	References
1	*ywaC*	GTP pyrophosphokinase	ppGpp synthase (putative)	Petrackova et al. (2010)
2	*proC, proA*	Pyrroline-5-carboxylate reductase, gamma-glutamyl phosphate reductase	Proline biosynthesis	Goswami et al. (2022)
3	*cysW*	Sulfate transport system permease protein	Sulfate/thiosulfate import	Goswami et al. (2018)
4	*ydbD*	Manganese-containing catalase	-	Price et al. (2001)
5	*ydaG*	General stress protein 26	Stress response	Current study
6	*gbsB*	Alcohol dehydrogenase	osmoprotection	Lawrance et al. (2021)
7	*aspA*	Aspartase	reversible deamination of the amino acid L-aspartic acid	Hu et al. (2010)
8	*gadA*	Glutamate decarboxylase	Converts glutamate to gamma-aminobutyrate (GABA)	Liu et al. (2015)
9	*opuA*	ABC transporter	Glycine-Betain uptake system	Li et al. (2023)
10	*spoOM*	Sporulation control protein	Sporulation	Vega-Cabrera et al. (2018)
11	*gbsA*	Glycine betaine aldehyde dehydrogenase	Biosynthesis of glycine betain	Lawrance et al. (2021)
12	*yknXc*	ATP-binding cassette transporter-like protein	Transporter	Petersohn et al. (2001)
13	*ctc*	Probable ribosomal protein	Function in translation	Gardan et al. (2003)
14	*clpC*	Class III stress response-related ATPase	Stress tolerance	Wozniak et al. (2012)
15	*csbC*	Metabolite transport protein homolog YwtG	Protection against stress	Hoper et al. (2005)
16	*yraA*	Intracellular proteinase I PfpI	detoxification of methylglyoxal	Thackray and Moir (2003)
17	*mrpB*	Na1/H1 antiporter BH1318	sodium export/ pH homeostasis	Haja and Adams (2021)
18	*sacC*	Levanase	Exo-fructosidase to produce free fructose	Velazquez-Hernandez et al. (2011)
19	*F1–F0–ATPase*	F1–F0–ATPase proton pump	Efflux the protons from intracellular environment to maintain the pH homeostasis	Kuhnert et al. (2004)
20	*recA*	RecA protein	DNA repair and SOS response during stress	van der Veen and Abee (2011)
21	*uvrA*	uvrA protein	Repair DNA damage during stress	Hanna et al. (2001)
22	*dnaK*	Hsp70 protein	Prevent protein misfolding during stress	Jayaraman et al. (1997)
23	*arcABC*	Arginine deiminase	Product of arcABC convert arginine to CO2 and ammonia to maintain cellular integrity during acidic stress	Rollan et al. (2003)
24	*ureABIEFGH*	Urease system	Urease gene cluster produce urease and by the process of ureolysis it produce ammonia and help to survive in acidic stress	Scott et al. (2002)
25	*gadC*	Glutamate/GABA antiporter	Export intracellular GABA and import glutamate into the cells	Yogeswara et al. (2020)
26	*ybaS*	Glutaminase	Glutamine was converted into glutamate and ammonia	Lu et al. (2013)
27	*arcD*	Hydrophobic polytopic membrane protein	Transport arginine in the intracellular environment	Guan and Liu (2020)
28	*nahA, nahG, nahH*	Key regulator of naphthalene degradation pathway	Break down the petroleum hydrocarbons, including anthracene, phenanthrene, toluene, and naphthalene	Koul et al. (2021)
29	*cpxA*	CpxA proteins	Control the regulation of fabA and fabB genes in response to acidic stress	Xu et al. (2020)
30	*cadA*	Lysine decarboxylase	Convert lysine to cadaverine by utilizing proton.	Merrell and Camilli (2002)
31	*cyo*	Cytochrome bo oxidase (CBO)	Electron transport chain	Kanjee and Houry (2013)
32	*ndh*	NADH dehydrogenase II (NDH-II)	-	Kanjee and Houry (2013)
33	*kdpABC*	ABC high-affinity potassium transport system	An inside-positive membrane potential through active influx of K+ to partially deflect the inward flow of protons	Chen et al. (2015)

Among the identified genes, *ydaG*, regarded as a general stress response gene, was selected to explore its role in acid tolerance. *ydaG* is one of the major general stress response genes that plays a significant role under different stress conditions (Petersohn et al., 1999). A previous transcriptomic study revealed a significant upregulation of *ydaG* transcript in the presence of acidic stress (Goswami et al., 2018). Through a series of experiments, we demonstrated the essential role of the *ydaG* gene in *P. megaterium* G18 tolerance to acidic environments. Targeted inactivation of the *ydaG* gene led to a significant reduction in survivability and growth under acidic pH conditions, highlighting its importance in acid resistance mechanisms. Growth characteristics revealed wild-type bacterium to grow normally at pH 7 and 4.5. Although the *ydaG* mutant grew normally at pH 7, the growth of the mutant was significantly reduced at pH 4.5, indicating the role of the *ydaG* gene in conferring acid resistance (Figures 5, 6). Furthermore, qRT-PCR analysis revealed a significant upregulation of the *ydaG* gene expression in response to acidic stress. The qRT-PCR analysis of wild-type and mutant cells at pH 7 and pH 4.5 at two different intervals (1 and 5 h) revealed that wild-type cells expressed *ydaG* at normal and acidic pH, however, the expression increased significantly at pH 4.5 at both time interval (Figure 7). The high expression of *ydaG* at acidic pH may be due to the acid shock, which facilitates the development of acid tolerance in bacteria when encountered with an acidic environment. The *ydaG* gene was reported to induce under heat, salt, and ethanol stress (Petersohn et al., 2001); its role in conferring acid resistance has not been previously reported. Sequence analysis and functional annotation of the gene point to its involvement in the synthesis of general stress proteins, especially those belonging to the pyridoxamine 5′-phosphate oxidase family. Although microscopic investigations show no appreciable effect on cellular shape, the inhibition of growth of the *P. megaterium* G18 in acidic pH 4.5 indicates its potential role in aiding resistance under acidic conditions. This finding highlights the potential biotechnological significance of *ydaG* in creating acid-resistant microbes for various uses, in addition to expanding our knowledge of the genetic pathways underpinning acid tolerance in microbial systems.

## 5 Conclusion

The genomic analysis of acid-tolerant bacteria offers valuable insights into the genetic basis of microbial adaptation to acidic environments. By elucidating the role of key genes such as *ydaG*, we enhance our understanding of microbial stress response mechanisms and their biotechnological potential. Moving forward, further research in this area promises to unlock new opportunities for the development of robust and resilient microbial systems for diverse applications.

## Data availability statement

The datasets presented in this study can be found in online repositories. The names of the repository/repositories and accession number(s) can be found at: the whole genome sequencing raw data of *Priestia megaterium* G18 have been deposited in NCBI under the BioProject accession number PRJNA785078 and Biosample accession number SAMN23526816.

## Author contributions

DS: Writing – review & editing, Methodology. PC: Data curation, Formal analysis, Investigation, Visualization, Writing – original draft, Writing – review & editing. VR: Data curation, Formal analysis, Investigation, Visualization, Writing – original draft, Writing – review & editing. SS: Writing – review & editing, Methodology, Formal analysis. BKS: Funding acquisition. MB: Writing – review & editing, Supervision, Conceptualization.

## Footnotes

^1^ https://pymol.org/2/

^2^ www.ncbi.nlm.nih.gov/gene

^3^ https://eu.idtdna.com/PrimerQuest

^4^ https://www.ebi.ac.uk/interpro/entry/InterPro/IPR012349/
