# Supplementary material for: Genomic analysis of acid tolerance genes and deciphering the function of ydaG gene in mitigating acid tolerance in Priestia megaterium
Source: Front Microbiol. 2024 Jun 20;15:1414777. doi: 10.3389/fmicb.2024.1414777 (PMC11222612; doi:10.3389/fmicb.2024.1414777)

Supplementary Material

Genomic analysis of acid tolerance gene and deciphering the role of *ydaG* gene in conferring acid tolerance in *Priestia megaterium*

Darshana Sharma[1†], Purna Bahadur Chetri[1†], Vipin Ranga[1], Subhajit Sen[1], Bidyut Sharma[1,2], Madhumita Barooah[1,2]

[1]DBT - North East Centre for Agricultural Biotechnology, Assam Agricultural University, Jorhat, Assam 785013, India.

[2]Department of Agricultural Biotechnology, Assam Agricultural University, Jorhat, Assam 785013, India.

†These authors contributed equally to this work and share first authorship

*** Correspondence:**
Dr. Madhumita Barooah
madhumita.barooah@aau.ac.in

Supplementary figure S1:- PCR amplification of *ydaG* gene. Lane 1, DNA ladder. Lane 2-3, PCR amplified product of *ydaG* gene.

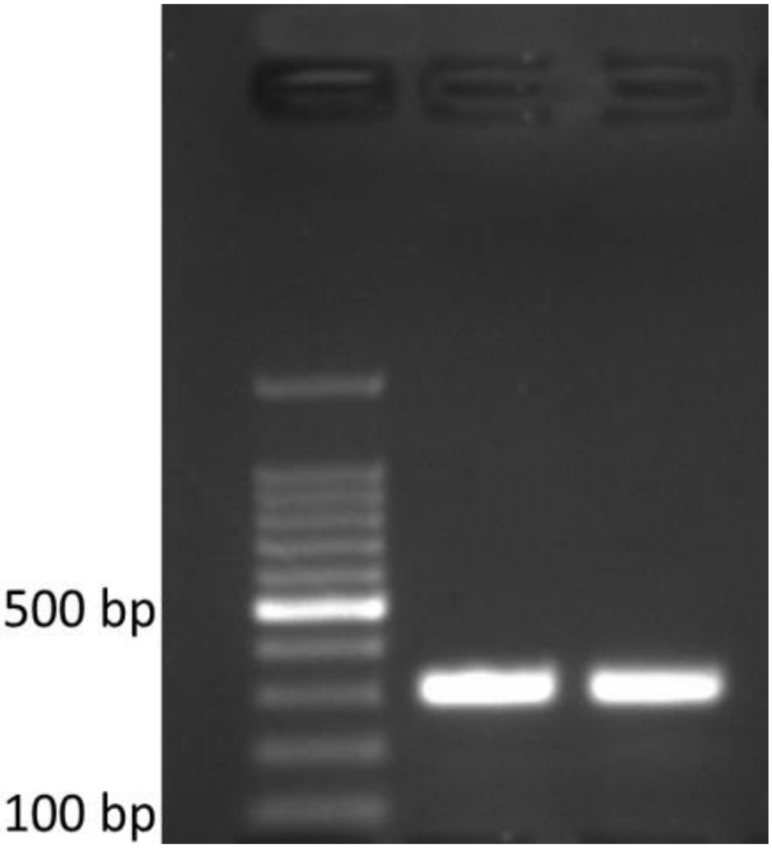

Supplementary figure S2:- PCR amplification of *ermAM* gene. Lane L, DNA ladder. Lane 1, negative clone, Lane 2-10, PCR amplified *ermAM* gene, which confirmed the integration of pMUTINydaG into the chromosome of *Bacillus megaterium* G18

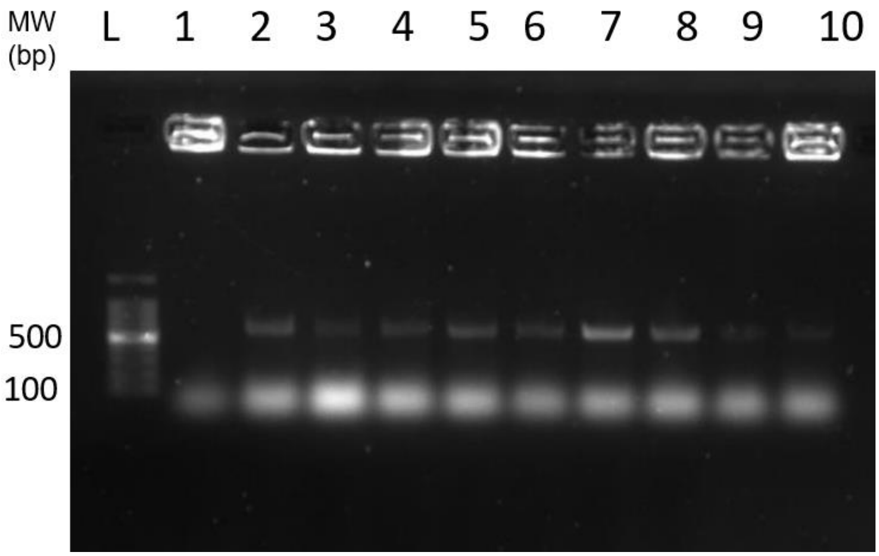

Supplementary figure S3:- PCR amplification of ydaG gene. Lane L, DNA ladder. Lane 1-7, No PCR amplification of ydaG gene, which confirms the targeted disruption of ydaG gene.

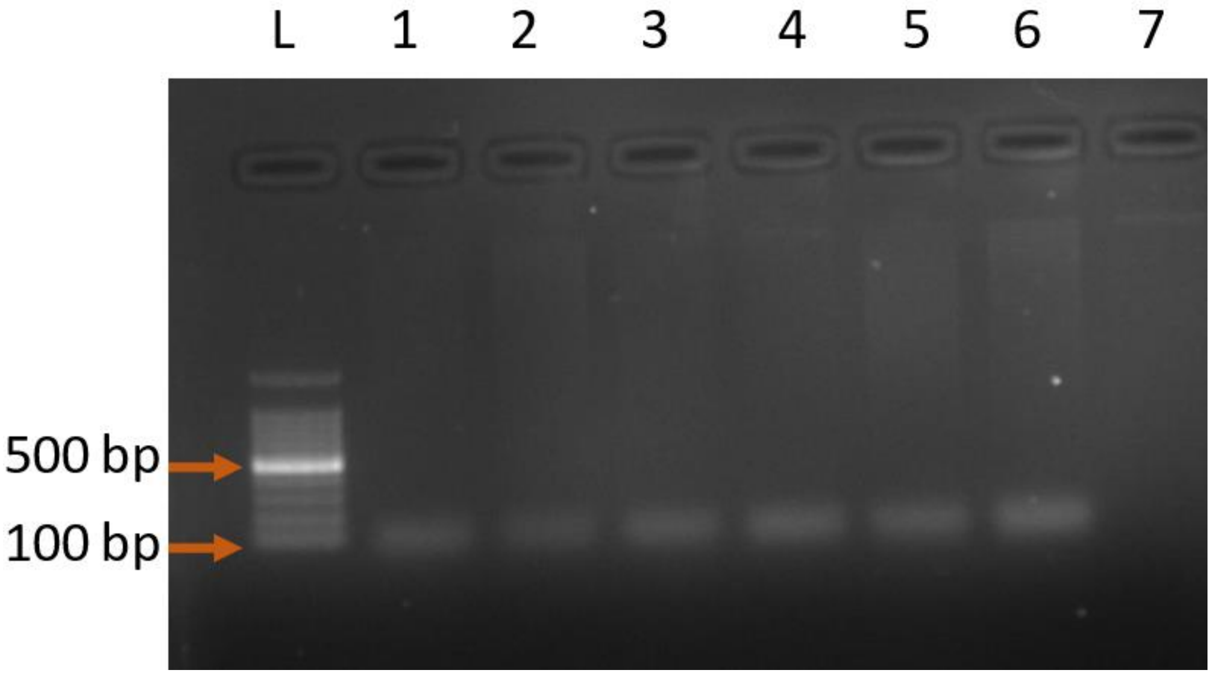

Supplementary figure S4:- PCR amplification of *ydaG* gene in wild type and mutant cells. Lane 1, DNA ladder. Lane 2-3, PCR amplification of *ydaG* gene of wild type cells at pH 7 and 4.5 respectively. Lane 4-5, No PCR amplification of *ydaG* gene of mutant cells at pH 7 and 4.5 respectively.

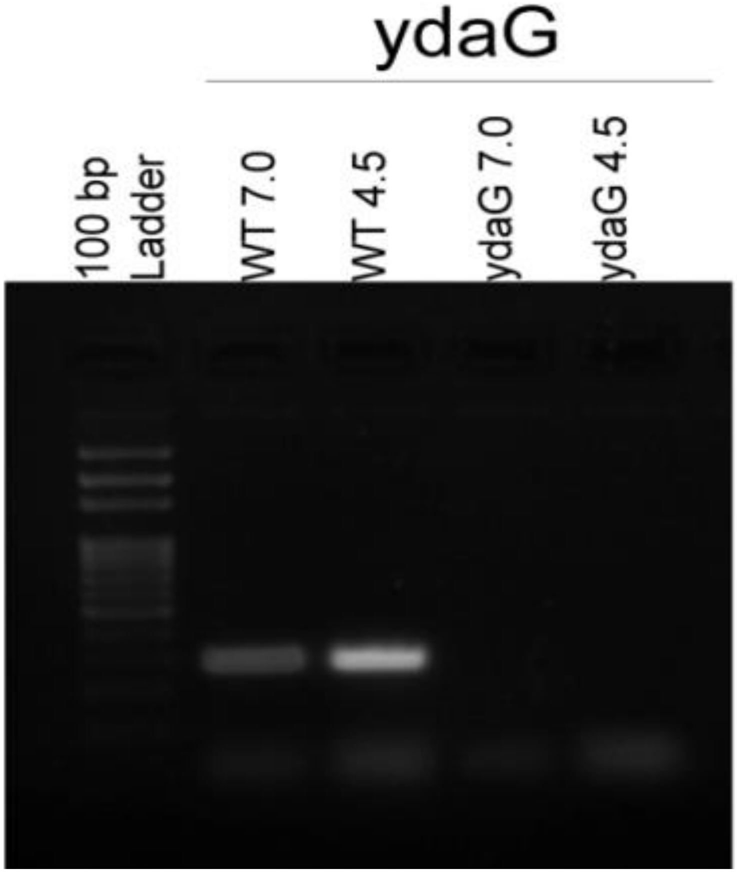

Supplementary figure S5: *Priestia megaterium* G18 colony characteristics. (A) In Luria agar (Himedia, India) medium yellowish white, circular, opaque, shiny, smooth or wrinkled, and mucoid or dry texture colonies were observed. (B) In HiCrome *Bacillus* agar (Himedia, India) medium yellow, smooth, circular, glistening, mucoid colonies were observed.

(A) **(B)**

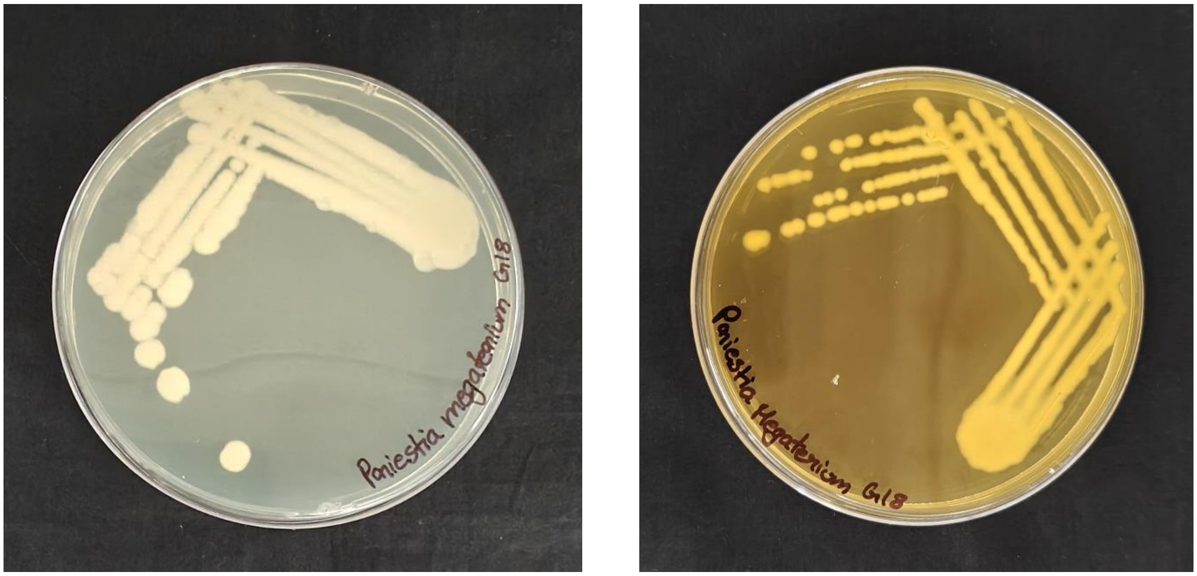

Supplement: Supplementary file 1 [file Presentation_1.pdf]
